# Editorial: Recent advances in the neuro-critical care of aneurysmal subarachnoid hemorrhage

**DOI:** 10.3389/fneur.2023.1230642

**Published:** 2023-06-30

**Authors:** Marvin Darkwah Oppong, Michael Veldeman, Jagos Golubovic

**Affiliations:** ^1^Department of Neurosurgery and Spine Surgery, University Hospital of Essen, Essen, Germany; ^2^Department of Neurosurgery, RWTH Aachen University Hospital, Aachen, Germany; ^3^Clinic of Neurosurgery Clinical Centre of Vojvodina, Faculty of Medicine, University of Novi Sad, Novi Sad, Serbia

**Keywords:** aneurysm, subarachnoid hemorrhage, neuro-intensive care, ventilation, delayed cerebral ischemia

Subarachnoid hemorrhage following the rupture of a brain aneurysm (aSAH) is a serious and often life-threatening condition. After timely securing of the aneurysm (i.e., surgical clip or endovascular coil), patient outcomes mainly depend on high quality neuro-intensive care and monitoring. The critical care management of aSAH patients is complex. Several neurological and medical conditions complicate the treatment, such as vasospasm with delayed cerebral ischemia (1), hydrocephalus (2), increased intracranial pressure, seizures (3), ventilator dependence, infections, blood pressure/electrolyte abnormalities (4), etc. As a result, critical care protocols for aSAH patients vary substantially from one institution to another and often lack standardization.

This Research Topic aimed to widen the knowledge and provide up-to-date critical care for the treatment of aSAH patients. The Research Topic currently includes seven articles from different fields, covering topics such as ventilation and sedation management, significance of laboratory markers, as well as prognosis and delayed cerebral ischemia (DCI).

Zhou et al. developed a nomogram to predict prognosis in poor-grade aSAH patients who underwent clipping. Laboratory markers to predict prognosis were addressed by Zhang et al. The authors connected the lactate albumin ratio with in-hospital mortality and incorporated it into a nomogram. In their review, Lasica et al. outlined the possible future utilization of metabolomics in monitoring aSAH patients.

One of the main factors that negatively impacts the wellbeing of aSAH patients is DCI, which is particularly devastating in patients with initial good conditions. Koopman et al. analyzed this subgroup by looking into the patients that developed severe DCI (with coma), and even though this group was small, in almost all patients, blood pressure instabilities were observed before DCI. The causes of DCI are multifactorial, with one being spreading depolarization. However, encephalographic monitoring is usually performed using a cortical grid electrode and remains quite invasive. Meinert et al. present a possibility to monitor this progenitor of DCI in a less invasive manner.

aSAH patients that require prolonged mechanical ventilation are candidates for tracheotomy. If it is predictable that long-term ventilation is necessary, such procedures could be performed as early as possible. However, it still remains unclear if, in this specific patient group, it might have a negative impact. Bini et al. analyzed patients with early percutaneous dilative tracheotomy. Even though a significant number experienced intracranial pressure peaks during the procedure, no negative impact on outcome was noted. Overall, no clear sedation/ventilation recommendations exist to date. Schmidbauer et al. therefore, performed a survey on the management of ventilation in aSAH patients among German neurointensivists.

This Research Topic in Frontiers in Neurology offers novel multiple insights into the complex pathophysiology of aSAH and its subacute complications. New diagnostic and therapeutic options have been assessed and proposed to improve general- and aSAH-specific intensive care treatment for these patients. Nonetheless, there is much potential for improvement, as many complications such as delayed cerebral ischemia still remain difficult to treat.

## Author contributions

MD drafted and wrote the first version of the manuscript. MV and JG critically revised the manuscript. All authors contributed to the article and approved the submitted version.
